# The effect of different root canal irrigations on the accuracy of apex locators: A systematic review

**DOI:** 10.4317/jced.62049

**Published:** 2024-12-01

**Authors:** Seyede Aye Shekarbaghani, Behnam Bolhari, Zohreh Khalilak

**Affiliations:** 1 Department of Endodontics, School of Dentistry, Baqiyatallah University of Medical Sciences, Tehran, Iran; 2 Department of Endodontics, Dental School/Dental Research Center, Tehran University of Medical Sciences, Tehran, Iran; 3 Endodontics, Tehran, Iran

## Abstract

**Background:**

For a successful root canal treatment, it is necessary to determine the correct working length. One of the most used tools to determine the length is: radiography and apex locator. The accuracy of APEX locator of different generations depends on various factors. Studies have reported various effects of root canal irrigations on Apex Locator accuracy. This review study collects the results of studies conducted in recent years so that dentists and specialists can choose the most appropriate root canal irrigation’s protocol with the least negative impact on the accuracy of Apex Locator.

**Material and Methods:**

This review article has been done in the form of a wide search in electronic databases such as MEDLINE, PubMed, BVS (Lilacs and BBO), Scopus, Web of Science, Cochrane Library and Embase in the period of 2010-2023. No language restrictions have been considered and the reference list of all related articles has been checked.

**Results:**

Out of 605 found articles, After removing duplicates and an initial screening, the full texts of only 25 articles are analyed and only 21 articles matched our inclusion criteria and their quality was assessed using modified CONSORT. This article is written based on the Preferred Reporting Items for Systematic Reviews and Meta‑Analyses protocol protocol. Based on the obtained results, use of hypochlorite alone, which has a negative effect on the accuracy of the apex locator due to its high electroconductivity,

**Conclusions:**

Use of a irrigation’s protocol such as the use of 17% Ethylenediaminetetraacetic acid (EDTA) after sodium hypochlorite or chlorhexidine (CHX) or 0.1% octinidine dohydrochloride (OCT) is suggested compared to the use of sodium hypochlorite alone.

** Key words:**Apex locator, irrigation solutions, sodium hypochlorite, Chlorhexidine, working length.

## Introduction

The success of root canal treatment (RCT) is highly dependent on accurately determining the correct root canal length, particularly from an anatomical perspective. The terminal point of the root canal is at the apical constriction (also known as the minor foramen), which serves as a crucial landmark for effective treatment (1). Studies examining periapical tissue after RCT have consistently shown that the best outcomes occur when treatment ends precisely at the apical constriction. Conversely, treatments extending beyond this point are associated with poorer prognoses. As such, locating the apical constriction is a critical step in clinical practice (2).

Various methods are available for determining root canal length, including radiography, tactile sensation, observation of bleeding, and knowledge of root canal morphology (2). Among these, the most commonly used approach is a combination of periapical radiographs and apex locators (3,4).

The first commercial electronic apex locator was introduced after the pioneering work of Custer and Sunada, who demonstrated the potential of using direct electrical currents to locate the apical foramen (5,6). Since then, apex locator technology has evolved through six generations, with each generation bringing improvements in accuracy and usability. The first two generations were highly sensitive to the canal environment and the irrigating solutions used during treatment, which limited their effectiveness (7).

The third generation of apex locators, which measures the ratio between two frequencies, has become widely accepted (2,3). The fourth generation, which uses mathematical calculations based on five frequencies, and the fifth generation, which compares electrical properties with mathematical calculations, also showed promising results (2,8). However, studies have indicated no significant difference in accuracy between the fourth generation and earlier models (1).

The latest advancement, the sixth generation of apex locators, incorporates the characteristics of canal irrigants, resulting in greater accuracy and reproducibility in root canal length determination (2). During the cleaning and shaping phases of endodontic treatment, irrigants play a vital role in disinfecting the canal. These irrigants must be effective against anaerobic and facultative microorganisms, capable of deactivating endotoxins, and non-toxic to the surrounding tissues (1,2,9).

Sodium hypochlorite (NaOCl) is the most commonly used irrigant due to its potent antibacterial properties and ability to dissolve necrotic tissue quickly. It is typically used in concentrations ranging from 0.5% to 6% (10). Another commonly used irrigant is chlorhexidine (CHX), a broad-spectrum antimicrobial agent available as both a gel and solution. Depending on its concentration, CHX can exhibit bacteriostatic or bactericidal effects (2,10,11).

Ethylenediaminetetraacetic acid (EDTA) is a chelating agent used to remove the mineral portion of the smear layer, with sodium hypochlorite often used afterward to remove the organic portion. EDTA is typically used at a concentration of 17%. (2,12,13) QMIX, a combination of EDTA, CHX, and a detergent, is an antimicrobial solution that not only removes the smear layer but also disrupts biofilms, showing superior efficacy over CHX in destroying *Enterococcus faecalis* biofilms (10).

Several factors can affect the accuracy of apex locators, including metal restorations, high electrolyte levels in blood or exudate within the canal, debris accumulation, and calcification (1,10). Other variables, such as pulp vitality, root resorption, canal diameter, and instrumentation, have also been reported to negatively impact apex locator performance (10,14). However, the influence of root canal irrigants on apex locator accuracy remains a topic of debate, with no definitive conclusions drawn so far (10).

Given the potential impact of irrigants on apex locator performance, a comprehensive and systematic review is warranted. This study aims to investigate the effect of different irrigants on the accuracy of apex locators, with the ultimate goal of improving clinical outcomes and optimizing root canal treatment procedures in practice.

## Material and Methods

This descriptive study is a systematic review according to the implementation method written based on the Cochrane Guideline for Systematic Review, and its reporting method is based on the principles of Prisma (15,16).

Eligibility criteria

The eligibility criteria of the included studies were developed with reference to participants, interventions, comparison, outcomes and study design (PICOS):

• Population: the apex locators, which is used to determine the canal length 

• Intervention: various root canal irrigants that improve the accuracy of the apex locators

• Comparison: not applicable

• Outcomes: The primary object of this systematic study is to investigate the clinical accuracy of Apex locators in the presence of various detergents.

The secondary aim of the study is to improve the clinical performance of apex locators and improve the quality of treatment.

This study is based on a systematic review of articles in MEDLINE, PubMed, BVS (Lilacs and BBO), Scopus, Web of Science, Cochrane Library and Embase, searching for keywords, i.e. a combination of the words irrigation solutions, working length, apex locator from January 1st, 2010 to December 31st, 2023.

The keywords for this are (sodium hypochlorite, apex locator, working length, endodontics, QMix, apical foramen, chlorhexidine, EDTA, irrigation, irrigation solutions, saline, Ethylenediaminetetraacetic acid, root canal, root canal length measurement) was used. The preferred search strategy for each database is shown in  Table 1. In addition, the reference lists of relevant articles and reports were checked manually.

The criteria for participation in this study were titles found with a combination of words such as irrigation solutions, working length and apex locator from the search engines MEDLINE, PubMed, BVS (Lilacs and BBO), Scopus, Web of Science, Cochrane Library and Embase. It was checked for thematic relevance and then relevance of the abstract.

Exit criteria

1. Articles whose full text is not available

2. Articles that did not have the desired information in their Review Article.

3. Gray literatures

References and “Related Articles” of the selected articles were also manually searched in MEDLINE, PubMed, BVS (Lilacs and BBO), Scopus, Web of Science, Cochrane Library and Embase. Articles matching the entry criteria were stored in resource management software (version 1.0.5X) End note and duplicate articles have been removed. Two researchers separately read the abstracts and titles of the selected articles and then extracted information such as publication year, study design, treatment details, type of intervention, and conclusion from the articles. The information extracted by two researchers is compared. If there was disagreement about the information extracted, a third researcher was consulted.

## Results

A total of 605 studies were initially identified via electronic search engines (PubMed, Scopus, and Cochrane Library databases…) and manual searches. Of the 605 articles, 204 duplicates

were discarded. After removing duplicates and an initial screening, the full texts of only 25 articles, all from electronic searches, were evaluated. Finally, 21 articles met the inclusion criteria and were included in this review.

**Figure 1:**
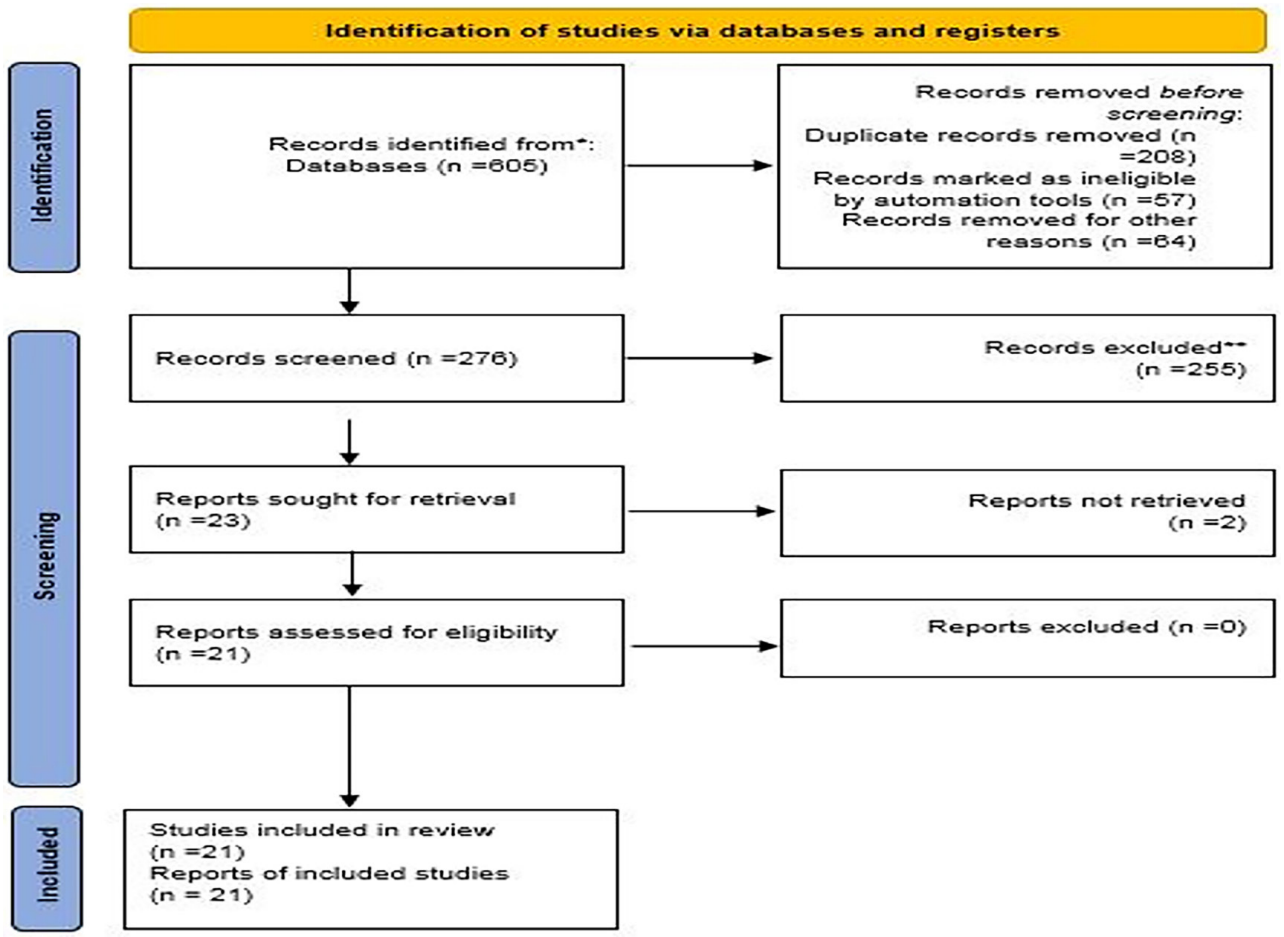
The PRISMA flow diagram.

21 selected articles and 18 articles were identified through electronic search and 3 articles were found in the reference list, (Fig. 1).

All studies examined the effect of irrigations on the accuracy of apex locators ( Table 2).

The following detergents were used in the intervention group.

NaOCl6%, NaOCl5%, NaOCl5.5%, NaOCl5.25%, NaOCl3%, NaOCl 2.5%, NaOCl 2%, NaOCl 2.125%, NaOCl 1%, H2O2 3%, CHX0.2%, Saline 0.9%, MTAD, QMIX, EDTA 17%, Xylol.

## Discussion

The accuracy of apex locators in endodontics is crucial for determining the length of the root canal, which in turn affects the overall success of endodontic treatments. Several studies and observations highlight various factors affecting the accuracy of these devices, especially in relation to file size and root canal irrigation content (10).

MacDonald emphasized the importance of using a file appropriate to the canal size for accurate apex locator readings (17). Ibrahim’s study indicated that using a smaller #10 K-File did not significantly impact clinical outcomes despite measurement errors, suggesting a certain level of tolerance (18). However, Heng’s research suggested that root canal irrigation content can affect apex locator accuracy, particularly noting that a small apical foramen might not be influenced by irrigation agents (19).

Studies have shown that apex locators can accurately display the location of the apical constriction, apical foramen, horizontal root fracture, and root resorption (10). Altunbas *et al*. suggested that apex locators are an accepted method for determining the location of perforations (20).

In Kaufman’s study, DENTAPORT (Morita Co, Japan) was more accurate than ROOT ZX (J. and MORITA CORP., KYOTO, JAPAN) in determining perforation length in the presence of NaOCl or EDTA (21). The new apex locator, whose performance depends on the comparison method, have shown good accuracy. Carneiro *et al*. showed in his study that TRI AUTO ZX (J, Morita Co, Kyoto, Japan) indicated the length of the canal to be shorter than the actual length, but this value was accepTable from a clinical perspective. A millimeter apex is more accurate than a 2mm apex (22). In the study of Erdemir the TRI AUTO ZX device with the AUTO REVERSE function was used and the vision in the environment of hypochlorite, H2O2, chlorhexidine, EDTA and ultracaine has an accuracy of 85%in determining the length in range of half a millimeter to, except in the environment of saline detergent which is 9% . this had an adverse impact on determining the length of this device (9).

The measurement accuracy of RAYPEX5 (VDW, Munich, Germany) and APEXDAL (Septodont, France) in chlorhexidine solution and gel and 2% hypochlorite was compared, and it was shown that 2% hypochlorite has a great influence on the measurement accuracy of RAYPEX5 and APEXDAL (23).

ASSUNCAO *et al*. believed that ROOT ZX MINI (J. MORITA CORP., KYOTO, JAPAN) has the same operating principles as ROOT ZX in dry and wet environments, but ROOT ZX had higher accuracy in the dry canal than in the wet canal with H2O2 (24).

The apex locator IPEX (NAKANISHI INC, TOCHIGI, JAPAN) has demonstrated similar accuracy to ROOT ZX in determining function length in the IN VIVO environment (1,25).

In the CHUKKA study, the accuracy of the apex locator VDW Gold (VDW, Munich, Germany) Root ZX Mini was evaluated in the medium chlorhexidine 2% and hypochlorite 3%, the results showed that the canal length in the chlorhexidine 2% is closer to the actual size. the result can be attributed to the different electrical conductivity of the solutions used. 3% hypochlorite showed a slightly shorter length, which could be due to the higher electrical conductivity of hypochlorite compared to chlorhexidine (8).

Hypochlorite is a highly electrically conductive detergent that can penetrate the dentinal tubules and lead to a reduction in electrical impedance. Electro conductive solutions are likely to produce better electrical exchange with the apical tissue (26)

Solutions with high electrical conductivity such as saline, anesthetic solution and hypochlorite may affect the accuracy of forth Apex generation (27).

1% hypochlorite with electrical conductivity (66 ms) and high alkaline pH (11.72) and EDTA with electrical conductivity (40 ms, pH (7.1)) caused shortening of the canal length with apex locator, CHX 2% with poor electrical conductivity (1ms) and (ph6.5) caused longer canal length in both ROOT ZX MINI and SYBRON MINI apexes. Similar results in the study by FAN *et al*. that electroconductive solutions significantly reduce the canal impedance and can lead to shorter measurements due to high electroconductivity (28).

 Although ROOT ZX MINI has been shown to have high performance accuracy in the studies, SYBRON MINI has higher performance accuracy in the detergent environment of 1% hypochlorite and 2% chlorhexidine as ROOT ZX MINI showed (27).

Many apex locators have measurement error, but ROOT ZX has proven to be a reliable apex locator. In the Meares study, ROOT ZX indicated canal length with accuracy 0.5 mm in 83% of cases (4) the Dunlup study, ROOT ZX had an accuracy of about half a millimeter in 82 to 96.02 percent of cases (29).

In Baruah’s study, apex locators (Root ZX Mini and Propex II) showed highest accuracy while working with 0.1% octinidine dohydrochloride (OCT) detergent and lowest accuracy when using heated hypochlorite 5%, reflecting the high electrical conductivity (66 milliseconds) of hypochlorite (30).

In this study, the order of accuracy of both apex locators in the presence of detergents was as follows (29): OCT>CHX>NaOCl>HEAT NaOCl

Similar to these results, Altunbas showed that Dentaport ZX has the lowest accuracy in presence of 2.5% hypochlorite detergent in perforated teeth. It is due to increase of free chlorine with increasing temperature (20).

In some studies, ELECTRONIC WORKING LENGTH (EWL) is greater than ACTUAL WORKING LENGTH (AWL) in weak electroconductivity solutions.

 VENTURI&BRESCHI reported that, reported measurements were inaccurate and inconsistent when using solutions with poor electroconductivity ratios (31).

 In both APEX locators ROOT ZX MINI and PROPEX II chlorhexidine and OCT caused Overestimation and hypochlorite and heated hypochlorite caused Underestimation of measurements were done (31).

furthermore, in the SINDREU study, they showed that ROOT ZX had higher accuracy in the presence of PENDEX detergent 0.12% and H2O2 than hypochlorite 5.25%, in this study, of the two APEX locators IPEX and ROOT ZX MINI were used, the measurements were significantly different, ROOT ZX MINI showed higher accuracy with hypochlorite detergent. 2.5% and chlorhexidine 2% compared to IPEX (1).

the study of VANDER, calcium hydroxide drug residues in the dentinal tubules reduced the permeability of tubules and caused errors in the treatment results. Ultrasonic activated hypochlorite was effective in removing calcium paste from the canal, particularly in the apical third of the root canal (32).

Shojaei *et al*. suggested that EDTA can be used as a chelating agent after NaOCl to remove calcium hydroxide drug residues from dentinal tubules. The final results showed that some amounts of calcium remained in the dentinal tubules, but these residues were not detected with the accuracy of the RAYPEX 6 apex locator (VDW, MUNICH, GERMANY) and ROOT ZX had no significant effect (12).

RAYPEX 6 and PROPEX II have higher measurement accuracy than IPEX II and ROOT ZX MINI in the QMIX environment (10).

Qmix, proposed as the final detergent after hypochlorite, is similar to hypochlorite 6% in terms of its effectiveness against E. FEACALIS. using Qmix detergent with Apex PROPEX II showed a performance accuracy of 94.7%, which showed higher accuracy compared to using this Detergent with APEX ROOT ZX MINI and IPEX II (10,26).

Riffat, who studied the effect of PREFLARING and various detergents on canal length determination by apex locator, used two apex locator models, RAYPEX 6 and ROOT ZX. Their results showed that the accuracy of RAYPEX 6 was not affected by detergents due to its MULTI FREQUENCY technology. This 6th generation apex locator is less sensitive to external factors such as the file taper and frequency with wich the file is used for pericoronal flaring. It was seeming that RAYPEX 6 showed the best results in the presence or absence of PREFLARING in a dry environment because in a dry environment it is easier to detect the canal resistance changing (7).

In the study by Kaufman *et al*. ROOT ZX and DENTAPORT were shown to have the required accuracy in dry environments.(21) same result seen in Kocak’s study, which showed that ROOT ZX MINI whose working principle is similar to ROOT ZX in dry environment had a much higher accuracy, although the concentration was 2.5% hypochlorite did not affect the accuracy of this device (10).

In contrast to these results, Vanturi’s study showed that the ROOT ZX was inaccurate and unstable in a dry environment where there are few conductive solutions. The accuracy of measuring the apex of locators is effective. (31) Altunbas believed that electroconductive materials such as alginate, agar and saline can simulate the clinical environment, and that leakage of some of these materials into the foramen results in excessive readings (20).

The results of the articles showed that there are variables such as the different degree of curvature of the tooth canal, the size of the apical foramen, which can affect the result, the agitating of the rinsing solution, the electrical conductivity of the detergents, the volume, concentration and temperature of the detergents and the using method of them affect the accuracy of the apex locators (1,10,33).

## Conclusions

According to the results of the articles, use of an irrigation protocol such as the use of EDTA after hypochlorite or CHX or OCT is recommended compared to the use of hypochlorite alone, which affects the accuracy of apex locators due to its high electroconductivity. With the advancement of technology, it is expected to use 6th generation Apex locators, which are less sensitive to external factors such as the file’s taper and number of used file for pericoronal flaring and shows high accuracy in a dry environment, should become more widespread.

## Figures and Tables

**Table 1:** Search Strategy for Each Database.

MEDLINE via PubMed	#1 (apex locator [Title/Abstract] OR working length [Title/Abstract] OR apical foramen [Title/Abstract] OR root canal [Title/Abstract] OR root canal length measurement [Title/Abstract])
	#2 (Endodontic irrigation [Title/Abstract] OR irrigation [Title/Abstract] OR irrigation solutions [Title/Abstract] OR chlorhexidine[Title/Abstract] OR CHX [Title/Abstract] OR QMix [Title/Abstract] OR Ethylenediaminetetraacetic acid [Title/Abstract] OR EDTA [Title/Abstract] OR saline [Title/Abstract] OR NaCl [Title/Abstract] OR QMix [Title/Abstract] OR sodium hypochlorite [Title/Abstract] OR NaOCl [Title/Abstract] OR endodontics [mesh])
	# 1 AND # 2 143
Scopus	# 1 TITLE-ABS (apex locator OR working length OR apical foramen OR root canal length OR root canal length measurement)
	#2 TITLE-ABS ("Endodontic irrigation "OR "Ethylenediaminetetraacetic acid" OR OR "EDTA" OR "saline" OR "NaCl" OR "sodium hypochlorite" OR "NaOCl" OR "irrigation" OR "irrigation solutions" OR "chlorhexidine" OR "CHX" OR "QMix"OR endodontics
	#1 AND #2 135
Web of Science	#1 TS5("apex locator" OR working length OR apical foramen OR root canal length OR root canal length measurement)
	#2 TS5("Endodontic irrigation" OR "Ethylenediaminetetraacetic acid" OR "EDTA" OR "saline" OR "sodium hypochlorite" OR "NaOCl" OR "irrigation" OR "irrigation solutions" OR "chlorhexidine" OR "CHX" OR "QMix" OR endodontics)
	# 1 AND # 2 108
BVS (Lilacs and BBO)	#1 (tw:("apex locator" OR working length OR apical foramen OR root canal length OR root canal length measurement)
	#2 (tw:("Endodontic irrigation" OR "Ethylenediaminetetraacetic acid" OR "EDTA" OR "saline" OR "NaCl" OR "sodium hypochlorite" OR "NaOCl" OR "irrigation" OR "irrigation solutions" OR "chlorhexidine" OR "CHX" OR "QMix" OR endodontics)
	#3 (tw:(Microbiology OR "Bacterial infection" OR Bacteria OR Microbiota)
	# 1 AND # 2 AND #3 74
Cochrane	#1 "apex locator" OR working length OR apical foramen OR root canal length OR root canal length measurement
	#2 "Endodontic irrigation" OR "Ethylenediaminetetraacetic acid" OR "EDTA" OR "saline" OR "NaCl" OR "sodium hypochlorite" OR "NaOCl" OR "irrigation" OR "irrigation solutions" OR "chlorhexidine" OR "CHX" OR "QMix" OR endodontics
	# 1 AND # 2 AND #3 71
Embase	#1 :("apex locator":ab,ti OR working length:ab,ti OR apical foramen:ab,ti OR 'post treatment':ab,ti OR root canal length:ab,ti OR root canal length measurement:ab,ti
	#2 'Endodonticirrigation':ab,ti OR 'Ethylenediaminetetraaceticacid' :ab,ti OR 'EDTA':ab,ti OR 'QMix':ab,ti OR'chlorhexidine':ab,ti OR 'CHX' /exp'saline':ab,ti OR 'NaCl':ab,ti /exp OR sodiumhypochlorite':ab,ti OR 'NaOCl'/exp OR 'irrigation':ab,ti OR 'irrigationsolutions':ab,ti OR 'endodontics':ab,ti
	#3 'microbiology' OR 'bacteriainfection'/exp OR 'bacteria'/exp OR microbiota:ab,ti
	# 1 AND # 2 AND #3 74

**Table 2:** Characteristics of studies related to effect of irrigations on apex locators.

Author	Title	Irrigator	Apex locator	Sample size	Conclusion
Vajpayee et al. (2023)	In vivo Study to Evaluate the Effect of Instrument Size on the Accuracy of Three Different Apex Locators when Various Irrigation Solutions are Used in Vital and Non-vital Teeth	3% NaOCl,2% CHX,17% EDTA	ROOT ZX	200 of the patients' teeth (100 vital and 100 nonvital first molar teeth	Even in the presence of irrigating solutions, electronic apex locator (EAL) can be utilized to calculate the working length with accuracy. The apex locators #15 k files, #10 k files, and #8 k files showed the most promising results.
Riffat et al. (2023)	The Effect of Coronal Pre-flaring and Type of Root Canal Irrigation on Working Length Accuracy Using Electronic Apex Locators	5.25% NAOCL,0.2% CHX	ROOT ZX RAYPEX 6	120 extracted single root	The dry medium with 6th generation EAL (Raypex 6) and 2% CHX with the 3rd generation EAL (Root ZX) showed the most accurate WL measurements
Karunakar et al. (2023)	Comparative evaluation of working length determined using integrated apex locator and root ZX mini under various irrigating solutions: An in vivo study	2.5% NAOCL,0.2% CHX,0.9% Salin	ROOT ZX mini CanalPro CL2i	30 single root anterior teeth	the irrigation solutions employed in this study had no impact on the performance of apex locators and radiographs
Cîmpean et al. (2023)	In Vitro Evaluation of the Accuracy of Three Electronic Apex Locators Using Different Sodium Hypochlorite Concentrations	2% NAOCL,5.25% NAOCL	ROOT ZX II ApeX ID Dual Pex	20 extracted single root	The best accuracy in working length determination was obtained by Root ZX II for 2% NaOCl solution and by Dual Pex for 5.25% NaOCl solution with no significant statistical difference when compared.
Topçuoğlu et al. (2023)	Evaluation of accuracy of an electronic apex locator in presence of sodium hypochlorite in primary teeth with and without resorption	1% NAOCL Non-NAOCL	ROOT ZX	32 root resorption & 32 without resorption teeth	NaOCl in the root canal affected the accuracy of the Root ZX mini in primary teeth with apical resorption, but not in teeth without resorption.
Dumani et al. (2022)	The Influence of MTAD and QMix on the Accuracy of Electronic Apex Locator in Locating Simulated Perforations	2.5% NAOCL 2% CHX MTAD QMix 0.9%Salin	Raypex6	25 extracted single root premolars	The most accurate electronic measurements of artificial perforation were obtained under dry conditions or with NaCl.
Chukka et al. (2020)	Efficiency of an Integrated Apex Locator in Determining Working Length in Various Irrigating Solutions: An In Vivo Study	3% NAOCL 2% CHX	ROOT ZX mini VDW gold	40 teeth	Both the apex locators were equally effective in determining WL at 0.5 mm from the apex in presence of irrigating solutions, that is, NaOCl and chlorhexidine.
Bilaiya et al. (2020)	Comparative Evaluation of Accuracy of Ipex, Root Zx Mini, and Epex Pro Apex Locators in Teeth with Artificially Created Root Perforations in Presence of Various Intracanal Irrigates	5% NAOCL 2% CHX 17% EDTA	ROOT ZX mini IPEX Epex pro	30 extracted single root	Most accurate measurement was seen in dry canals for all three EALs. . No significance difference was observed in iPex and Epex Pro Apex locator, and between NaOCl and CHX, CHX and EDTA.
Shojaee et al. (2019)	Influence of calcium hydroxide residues after using different irrigates on the accuracy of two electronic apex locators: An in vitro study	5.5% NAOCL Salin	ROOT ZX Raypex6	80 extracted single root	No statistically significant differences were observed between the two apex locators after Ca(OH)2 paste removal with different irrigates.
Marek et al. (2019)	The influence of two forms of chlorhexidine on the accuracy of contemporary electronic apex locators	2% CHX solution 2% CHX gel 2% NaOCl	Raypex5 ApexDal	29 single-rooted vital human teeth (incisors and upper second premolars)	The apex locators Raypex 5 and ApexDal locate with the highest accuracy the anatomical foramen of the root containing CHX in the gel or in the solution form than in canal containing the NaOCl.
Baruah et al. (2018)	Comparative Evaluation of Accuracy of Two Electronic Apex Locators in the Presence of Contemporary Irrigants: An In vitro Study	2% CHX 5% NAOCL (heated & nonheated) 0.1% OCT	ROOT ZX mini Propex II apex	80 extracted single root	Electronic length measurements were shorter with heated and nonheated 5% NaOCl and longer with 0.1% OCT and 2% CHX for both the electronic apex locators.
Bolbolian et al. (2018)	In Vitro Evaluation of the Accuracy of the Root Zx in the Presence of Naocl 2.5% and Chlorhexidine 0.2%	2.5% NAOCL 0.2% CHX	ROOT ZX	20 extracted PREMOLAR	Root zx can accurately determine the apical constriction in presence of both NaOCl 2.5% and chlorhexidine 0.2%.
Koçak et al. (2016)	Influence of QMix Irrigant on the Accuracy of Four Different Electronic Apex Locators	2.5% NAOCL QMix	ROOT ZX Raypex6 iPex II Propex II	19 mandibular incisor teeth	Root ZX mini was found more accurate under dry Condition. Raypex 6 demonstrated more accuracy in the presence of QMix when compared with NaOCl. iPex II showed similar measurements with all tested solutions. Propex II was more accurate in the presence of QMix. All devices can be considered reliable when used with QMix
Altunbas et al. (2016)	The Influence of Various Irrigants on the Accuracy of 2 Electronic Apex Locators in Locating Simulated Root Perforations	2.5% NAOCL 17% EDTA 0.9 % Salin	Dentaport ZX	20 extracted straight single root teeth	The Dentaport ZX was more accurate compared with the Rootor in the presence of different irrigants. The content of the root canal affected the accuracy of both EALs. The most accurate measurements were obtained in dry canals
Khattak et al. (2014)	A comparative assessment of the accuracy of electronic apex locator (Root ZX) in the presence of commonly used irrigating solutions	2.5% NAOCL 0.2% CHX Salin	ROOT ZX	60 extracted single root	The accuracy of EL measurement of Root ZX AL was consistently high in the presence of 0.2% chlorhexidine, normal saline and 2.5% sodium hypochlorite.
DURAN-SINDREU et al. (2013)	In vivo evaluation of the iPex and Root ZX electronic apex locators using various irrigants	2.5% NAOCL 2% CHX	IPEX ROOT ZX	32 extracted single root	Although IPEX showed less accuracy than ROOT ZX, but both were affected by detergents.
Paras mull et al. (2012)	Comparison of accuracy of two electronic apex locators in the presence of various irrigants: An in vitro study	1% NAOCL 2% CHX 17% EDTA 0.9% Salin	ROOT ZX SybronEndo	60 extracted single root	The electronic length measurements were shorter with 1% NaOCl, whereas longer with 2% CHX for both the devices. Sybron Mini was more accurate with 1% NaOCl and 2% CHX than Root ZX.
Erdemir et al. (2007)	The influence of irrigating solutions on the accuracy of the electronic apex locator facility in the Tri Auto ZX handpiece	5.25% NAOCL 0.2% CHX 17% EDTA 3% HO2O2 0.9 % Salin DRY	Tri auto ZX	140 single root teeth with mature apices	Tri Auto ZX gave reliable results with all irrigating solutions apart from in the presence of 0.9% saline.
Ebrahim ak et al. (2006)	The effects of file size, sodium hypochlorite and blood on the accuracy of Root ZX apex locator in enlarged root canals: an in vitro study	6% NAOCL	ROOT ZX	36 extracted lower premolar teeth	presence of NaOCl, the Root ZX was highly accurate even when the file was much smaller than the diameter of the canal. The agar model was effective and suitable for testing EALs in vitro.
Kaufman et al. (2002)	Accuracy of a new apex locator: an in vitro study	3% NaOCl saline 0.2%Chlorhexidine 17% EDTA Xylol	Root ZX Bingo 1020	120 extracted multi- and single-rooted teeth	In the presence of EDTA and saline, measurements were closer to the AL, whilst those carried out in dry canals or in the presence of Xylol were shorter.
Meares et al. (2002)	The Influence of Sodium Hypochlorite Irrigation on the Accuracy of the Root ZX Electronic Apex Locator	2.125% NAOCL 5.25% NAOCL	ROOT ZX	40 extracted single root	Root ZX is not adversely affected by the presence of sodium hypochlorite

## Data Availability

The datasets used and/or analyzed during the current study are available from the corresponding author.
